# New Neutralizing Epitope Exposed on the Domain II of Tick-Borne Encephalitis Virus Envelope Glycoprotein E

**DOI:** 10.3390/v15061256

**Published:** 2023-05-26

**Authors:** Andrey Matveev, Yana Khlusevich, Irina Kozlova, Leonid Matveev, Lyudmila Emelyanova, Artem Tikunov, Ivan Baykov, Nina Tikunova

**Affiliations:** 1 Institute of Chemical Biology and Fundamental Medicine, Siberian Branch of the Russian Academy of Sciences, 630090 Novosibirsk, Russia; guterus@gmail.com (A.M.);; 2 Federal State Public Scientific Institution “Scientific Centre for Family Health and Human Reproduction Problems”, Siberian Branch of Russian Academy of Sciences, 664003 Irkutsk, Russia

**Keywords:** tick-borne encephalitis virus, protective antibody, flavivirus, protein E, epitope mapping

## Abstract

*Orthoflavivirus encephalitidis*, formerly tick-borne encephalitis virus (TBEV), belongs to the *Orthoflavivirus* genus. TBEV is transmitted by tick bites and infection with TBEV can lead to serious disorders of the central nervous system. In this study, a new protective monoclonal mouse antibody (mAb) FVN-32, with high binding activity to glycoprotein E of TBEV, was selected and examined in post exposure prophylaxis in a mouse model of TBEV infection. BALB/c mice were injected mAb FVN-32 at doses of 200 μg, 50 μg, and 12.5 μg per mouse one day after a TBEV challenge. mAb FVN-32 showed 37.5% protective efficacy when administered at doses of 200 μg and 50 μg per mouse. The epitope for protective mAb FVN-32 was localized in TBEV glycoprotein E domain I+II, using a set of truncated fragments of glycoprotein E. Additionally, the target site recognized by mAb FVN-32 was defined using combinatorial libraries of peptides. Three-dimensional modeling revealed that the site is dspatially close to the fusion loop, but does not come into contact with it, and is localized in a region between 247 and 254 amino acid residues on the envelope protein. This region is conserved among TBEV-like orthoflaviviruses.

## 1. Introduction

Flaviviruses are vector-borne viruses that can appear unexpectedly in humans and cause serious diseases. *Orthoflavivirus encephalitidis*, formerly known as tick-borne encephalitis virus (TBEV), is a single-stranded (+)RNA virus belonging to the *Orthoflavivirus* genus. Although more than 65% of TBEV infection cases in humans occur in an asymptomatic form, tick-borne encephalitis (TBE) can manifest with neurological disorder that may lead to meningitis, meningoencephalitis, and encephalomyelitis [1]. TBEV is transmitted by the bite of several species of ticks belonging to the *Ixodes* genus; however, other routes, such as the consumption of unpasteurized milk and milk products obtained from infected animals, remain important [1,2].

Despite numerous studies, there are currently no licensed specific drugs for the treatment of TBE in European countries. To treat patients with TBE, only supportive and symptomatic care is commonly used [1]. FSME-Bulin, a specific anti-TBE serum immunoglobulin, was previously applied as prophylaxis after exposure with TBEV; nevertheless, the use of FSME-Bulin has been discontinued because of its suspected ability to mediate TBE. In Russia and the Republic of Kazakhstan, specific anti-TBE serum immunoglobulin is still used for post-exposure prophylaxis and treatment of severe TBE, as the largest number of cases of fatal TBE have been recorded in these countries [1].

Currently, several vaccines are used to prevent TBE in European countries, China, and some post-Soviet states. The administration of these vaccines demonstrates their efficacy, safety, and high immunogenicity. The coverage of vaccination differs between different endemic territories of Eurasia. It was shown that low vaccination rates are associated with significantly higher incidence of TBE [1]. Furthermore, several cases of TBE vaccine breakthrough have been noticed in European countries [3,4,5]. Cases of vaccine breakthrough and the unavailability of approved safe and specific anti-TBE drugs stimulate the development of improved vaccines.

The most immunogenic protein of TBEV is glycoprotein E, which is the exposed structural protein in the virion of TBEV. Glycoprotein E is involved in the assembly of virus particles and plays a key role in the entry of the virus into the host cells. This protein comprises three domains: DI, DII, and DIII [6]. DI contains the N-glycosylation site, whereas the fusion loop is located in DII [7]. The DIII domain has an immunoglobulin-like structure [6] and is the most likely candidate for interactions with cellular receptors. Importantly, the most effective neutralizing antibodies that appeared during the infection are directed against DIII [8,9], whereas in vaccinated persons, neutralizing antibodies are mostly elicited against DII [10]. It has been suggested that anti-DII antibodies and some anti-DIII antibodies can be responsible for the antibody-dependent enhancement (ADE) of some flavivirus infections [10,11,12,13,14]. The main reason for the ADE of flavivirus infections is due to the ability of anti-glycoprotein E antibodies to bind the virus and interact with Fcγ receptors (FcyR) presented on immune cells. This leads to infection of immune cells, which enhances flavivirus infection [15,16].

An additional FcγR-independent mechanism of ADE for TBEV infection has been described recently [11]. This mechanism is released by conformational changes in the fusion loop of glycoprotein E after binding with an antibody. This allows TBEV to attach more effectively to target cells, including FcγR-negative cells [11]. ADE has been demonstrated by antibodies binding glycoprotein E of different orthoflaviviruses in in vitro experiments [17,18]. Therefore, the search and mapping of new epitopes recognized by neutralizing, especially weakly neutralizing anti-glycoprotein E monoclonal antibodies (mAbs), is of interest for vaccine development.

In this study, we describe a new epitope recognized by mAb FVN-32, which is derived from mice that were consecutively immunized with recombinant protein E and TBEV. In in vivo experiments, it was shown that mAb FVN-32 is weakly protective and its epitope is localized in a region from 247 to 254 amino acid residues (aa) in DII, which is conserved among TBEV-like orthoflaviviruses, and does not contact the fusion loop. The data obtained should be taken into account when developing and evaluating improved vaccines.

## 2. Materials and Methods

### 2.1. Animals, Virus, and Cells

The strain Sofjin, which belongs to the Far Eastern subtype of TBEV, was received from the FSPSI «SC FHHRP» repository (Collection # 478258, http://www.ckp-rf.ru (accessed on 30 April 2023), Irkutsk, Russia). BSL-3 protocols were applied in experiments with live TBEV.

Female mice (line BALB/c) were acquired from the animal care facility of FSRC VB “Vector”. Mice were treated in a way described in recommendations for the use of laboratory animals used for experiments (EU Directive 2010/63/EU). Approval of the bioethics committee of FSPSI «SC FHHRP» (Irkutsk, Russia) was received to perform all experiments with mice.

*Escherichia coli* BL21(DE3), *E. coli* TG1, *E. coli* HB2151, and *E. coli* ER2738 cells were obtained from the Collection of Extremophile Microorganisms and Type Cultures of ICBFM SB RAS.

### 2.2. Expression of Recombinant Proteins

Plasmids encoding fragments of DI and DII—namely, rED2ΔA (1–141 aa), rED2ΔB (aa 130–195), rED2ΔC (aa 189–230), and rED2ΔD (aa 231–260)—were constructed based on the sequence of the TBEV glycoprotein E (strain Sofjin-Ru JN229223). Plasmid pHEN- rED(1–2) was used as a template to amplify DNA fragments encoding truncated variants of DII protein E rED2ΔA (1–141 aa), rED2ΔB (aa 130–195), rED2ΔC (aa 189–230), and rED2ΔD (aa 231–260). The list of oligonucleotides used for this PCR is given in Table 1. Plasmid pET32a and PCR fragments encoding truncated variants of DII protein E were digested with *Bam*HI and *Eco*RI and then ligated. Plasmids pET32a-rED2ΔA, pET32a-rED2ΔB, pET32a-rED2ΔC, and pET32a-rED2ΔD were used to transform *E. coli* BL21(DE3) cells and were cultivated onto agar containing isopropyl-β-D-1-thiogalactopyranoside (20 μg/mL) and ampicillin (50 μg/mL).

Plasmids encoding truncated analogs of TBEV glycoprotein E (Sofjin-Ru JN229223) were constructed with the use of vector pHEN-2 as published previously [19]. Obtained truncated proteins correspond to glycoprotein E aa: rE, aa 1–397; rED(1–2) (domains I+II), aa 1–299; and rED3_301 (domain III), aa 301–395 [20]. Proteins rE and rED(1–2) were produced in *E. coli* TG1 cells, whereas *E. coli* HB2151 cells were used for production of the protein rED3_301.

### 2.3. Mouse Immunization and mAbs Selection

To develop antibodies against TBEV glycoprotein E, recombinant protein rE was used for the first immunization. To prepare the emulsion, 40 μg of recombinant protein rE (aa 1–397) in phosphate buffer saline (PBS), pH 7.4, was suspended with an equal volume of Freund’s complete adjuvant. The prepared emulsion was used for subcutaneous administration to 12–14-week-old female BALB/c mice (21–27 g). Each mouse was additionally infected with 30 plaque-forming TBEV units, Sofjin strain, two and four weeks after the first immunization. The final immunization was performed two weeks after the second infection and mice were immunized with rE (aa 1–397) in PBS without adjuvants. Three days after the final immunization, mouse splenocytes were obtained. Then, fusion of SP2/0 myeloma cells with the obtained splenocytes was performed using PEG 2000 (Roche, Basel, Switzerland) in accordance with the protocol described previously [21]. Hybridoma clones producing antibodies against glycoprotein E were selected by an anti-rE mAb titer in supernatants using ELISA. The limiting dilution method was applied two times to clone the hybridoma-producing mAb FVN-32 (IgG1 subclass with the kappa-type light chains).

### 2.4. Production, Purification, and Evaluation of the mAb

Hybridoma cells (2 × 10^6^) were used to produce mAb FVN-32. The cells were injected intraperitoneally into two 20-week-old BALB/c mice. MabSelect protein A resin (GE Healthcare, Chicago, IL, USA) for affinity chromatography was applied to purify mAb FVN-32 from mouse ascites according to the mice IgG1 purification protocol. After purification, mAb FVN-32 was concentrated using Sartorius Vivaspin Turbo 4 50 kDa (Sartorius, Gottingem, Germany). Buffer (50 mM Tris-HCl, pH 7.5, 150 mM NaCl, 0.05% NaN_3_) was used to store mAb.

The affinity constant of mAb FVN-32 was determined using the protocol previously described by Levanov et al. [22]. Recombinant glycoprotein E of TBEV was sorbed in 96-well microtiter plates (200 ng per well) at 4 °C overnight and then blocked with 5% skim milk for one hour at 37 °C. After incubation, the plates were washed with PBS. mAb FVN-32 was serially diluted in PBS containing 0.05% Tween-20 (PBST) and added to wells. Then, plates were placed in a thermostat for one hour at 37 °C. Immune complexes were detected with an alkaline phosphatase-conjugated goat anti-mouse IgG (Sigma Aldrich, St. Louis, MO, USA) and visualized with para-nitrophenylphosphate. The sigmoid curves were drawn to view the interaction of OD_405_ versus lg(DM). DM represents the dilution multiples of the antibody. Binding affinities were calculated according to the following equation:

$$OD_{405}\left(A_{0}\right)=N\times \left(A_{0}+\frac{b_{0}}{V}+K_{d}-\sqrt{{\left(A_{0}+\frac{b_{0}}{V}+K_{d}\right)}^{2}-4\times A_{0}\times \frac{b_{0}}{V}}\right),$$

where *OD*_405_ is the optical absorbance of solution in the microplate well, *A*_0_ is the total concentration of antibody in solution, *b*_0_ is the total concentration of the recombinant glycoprotein E, *K_d_* is the dissociation constant of the antigen–antibody complex on the surface, *V* is the total volume of solution in the microplate well, and *N* is the normalization factor, which is determined by the experimental conditions and does not depend on the antibody concentration. Regression analysis was performed using the Origin 7.0 software (OriginLab Corporation, Northampton, MA, USA).

### 2.5. Animal Studies

TBEV strain Sofjin at a dose of 5 fifty percent real lethal doses (RLD50) was intraperitoneally injected (0.2 mL) in 2 week-old BALB/c mice (10–14 g). The dose approximately corresponds to the minimum dose of TBEV in a tick in nature. BSL-3 protocols were applied in all experiments with live TBEV.

Mice were administered an intramuscular (thigh muscles of the hind limb) injection of mAb FVN-32 in a volume of 100 μL one day after infection with TBEV. Eight mice formed each experimental group; the control group of non-treated mice consists of six animals. Observation for the animals continued for three weeks after infection. Reed–Muench method was used to count fifty percent real lethal doses RLD50 [23]. The RLD50 was determined in each experiment.

### 2.6. Western Blot Analysis

Centrifugation of *E. coli* cells that produce truncated analogous of glycoprotein E was performed after 4 h of cells growth with IPTG. Lysis buffer (50 mM Tris-HCl, pH 6.8, 4% sodium dodecyl sulfate, 200 mM dithiothreitol) was added to cell precipitates, then precipitates were fractionated by 12.5% SDS-PAGE and transferred to nitrocellulose membrane. Skim milk diluted in PBST was used as a blocking agent. Then, membranes were incubated at 37 °C for one hour with mAb FVN-32 (5 μg/mL); incubation with anti-TBE mice sera was used as a positive control. Goat anti-mice IgG conjugated with horse-radish peroxidase (Sigma Aldrich, St. Louis, MO, USA) was used to reveal antigen–antibody complexes formed. 4-Chloro-1-naphthol (Sigma-Aldrich, St. Louis, MO, USA) was applied to visualize the complexes.

### 2.7. Epitope Mapping

The phage display method was performed for epitope mapping using the phage display libraries PhD-C7C and PhD-12 (New England Biolabs, Ipswich, MA, USA). Specific peptides were selected as described in Matveev et al. [20]. Non-specific mAb 3G11, belonging to the IgG1 sub-class with kappa-type constant region of light chains [24] was incubated with phage particles (10^11^) from each library. After depletion, phage particles were added to 96-wells plates with sorbed FVN-32 (200 ng per well) in PBS. After incubation at 37 °C for 60 min, unbound phages were removed. Elution of specific phages was performed with 100 mkg/mL mAb FVN-32 and eluted phages were used for the next rounds of biopaning. Only 20 ng per well of FVN-32 were used in the following biopaning rounds. *E. coli* cells (ER2738) were transfected by bacteriophages eluted after the second round of biopaning. Individual plaques were used to obtain individual bacteriophages. The phages were sequenced and tested in ELISA to bind FVN-32.

For indirect ELISA, 20 ng mAb FVN-32 was sorbed in 96-well plates. Unique selected phages were used at 10^10^ approximate concentrations. Bound phage particles exposing the peptide were detected with rabbit anti-M13-polyclonal antibodies. The immune complexes formed were detected by the conjugate of alkaline phosphatase and anti-rabbit antibodies, which were detected by para-nitrophenylphosphate (Roche, Basel, Switzerland). As a negative control, we used the detection of sorbed mAb FVN-32 with “wild-type” bacteriophage M13, which does not carry a peptide insertion on the surface of the protein p3.

To determine aa sequences of the FVN-32-specific peptides selected from library, the appropriate gene fragments encoding these peptides were amplified by PCR using oligonucleotides M13_pIII_F (5′-CTCTGTAGCCGTTGCTAC-3′) and M13_PIII_96 (5′-CCCTCATAGTTAGCGTAACG-3′). Then, the fragments were sequenced using the BigDye Terminator v3.1 cycle sequencing kit and the 3500 DNA Analyzer (Applied Biosystems, Foster city, CA, USA)

### 2.8. Statistics

Microsoft Excel software was used to analyze experimental data and to calculate mean values and standard error of the mean (SEM). Kaplan–Meier curves of survival were compared by the log-rank test. An online service (https://www.evanmiller.org/ab-testing/survival-curves.html (accessed on 30 April 2023)) was used to provide this calculating with statistical significance *p* < 0.05.

## 3. Results

### 3.1. MAb FVN-32 Generation and Characterization

Individual hybrid clones were screened for their binding with rE and the hybridoma clone specifically producing mAb FVN-32 was selected. The affinity constant of mAb FVN-32 assessed by ELISA was (1.42 ± 0.2) × 10^9^ M^−1^, indicating that mAb FVN-32 has nanomolar affinity. To determine the IgG class of mAb FVN-32, PCR fragments encoding the constant domain CH1 and the light chain constant domain were obtained and sequenced as described in Matveev et al. [25]. The heavy and light chains of mAb FVN-32 belong to the IgG1 class and kappa family, respectively.

### 3.2. Post-Exposure Administration of mAb FVN-32

**Figure 1:**
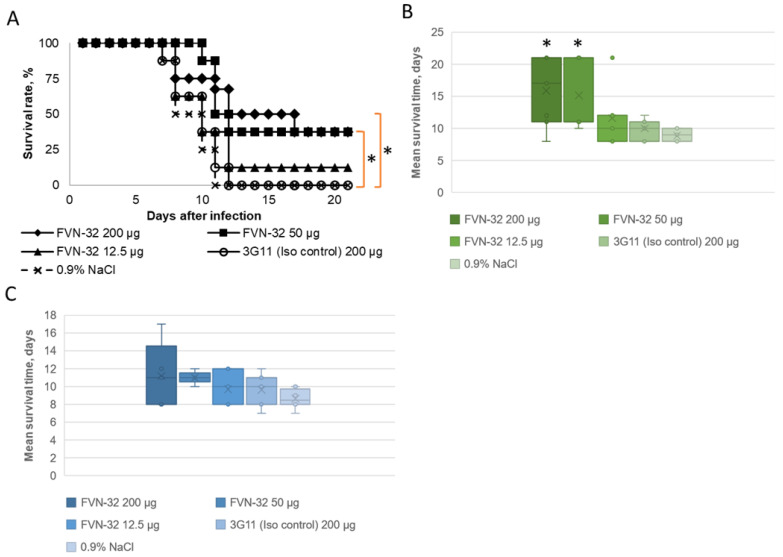
Efficacy of mAb FVN-32 in post-exposure prophylaxis. BALB/c mice (10 g) were treated i.v. with mAb FVN-32 or IgG1 mAb 3G11 at the indicated doses 24 h after injection of 5 RLD50 of TBEV, strain Sofjin. (**A**) Survival curves per treatment group (Kaplan–Meier curve). (**B**) Survival rates. (**C**) Mean survival time of mice excluding survivors. Results given are MST ± SEM, * *p* < 0.01 (log-rank test).

BALB/c mice were challenged with TBEV, strain Sofjin, by i.p. injection at a dose of 5 RLD50. After 24 h post infection, three groups of mice were injected i.m. with FVN-32 at doses of 200 μg/mouse, 50 μg/mouse, and 12.5 μg/mouse, respectively. Eight mice were administrated with the isotypic control mAb 3G11 [24], having IgG1/kappa constant domains of heavy and light chains, accordingly. Six untreated mice were negative controls. After administration of mAb FVN-32, the survival rate was 37.5% for the 200 μg/mouse and 50 μg/mouse, whereas no protection was observed when mAb FVN-32 was administered at a dose of 12.5 μg/mouse or the IgG1 isotype control mAb 3G11 was injected (Figure 1).

Since the antibody showed weak protective activity, the mean survival time of the animals, excluding survivors, was analyzed. Administration of mAb FVN-32 at all used doses did not decrease the mean survival time. It was shown that the mean survival time in the antibody-treated groups of mice was comparable to or better than that in the control group of untreated mice and the isotypic control group (Figure 1). These results indicated that administration of the mAb FVN-32 did not enhance infection caused by TBEV in the mouse model.

### 3.3. Neutralizing Epitope Mapping

**Figure 2:**
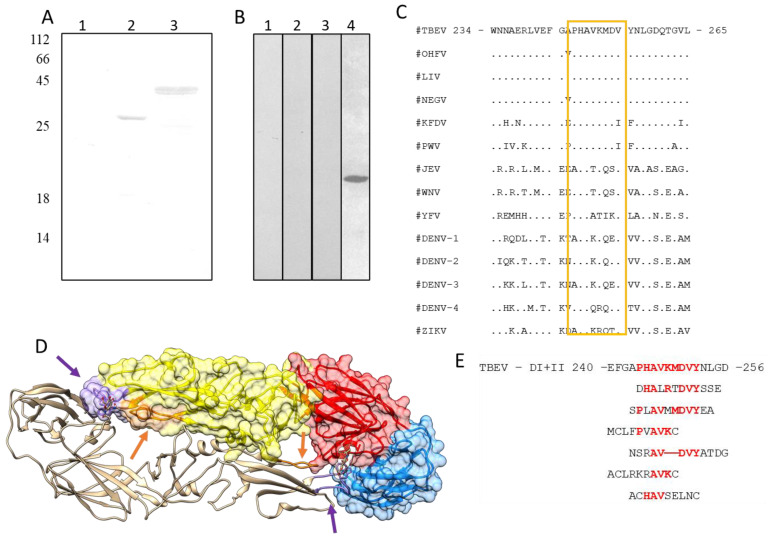
Epitope mapping of mAb FVN-32. (**A**) Western blot analysis of lysates of *E. coli* cells expressing rED3_301 (line 1), rED1+2 (line 2), and rE (line 3), which were fractionated by SDS-PAGE (12.5%) and revealed using mAb FVN-32. (**B**) Western blot analysis of lysates of *E. coli* cells expressing rED2delA (line 1), rED2delB (line 2), rED2delC (line 3), and rED2delD (line 4), which were fractionated and revealed as above. Protein mass markers in kilodaltons are represented at the left sides of a gel. (**C**) Alignment of the identified fragment (aa 240–260) in domain II of orthoflavivirus protein E. Abbreviations: OHFV—omsk hemorrhagic fever virus, LIV—looping ill virus, NEGV—Negishi virus, LGTV—Langat virus, PWV—Powassan virus, KFDV—Kyasanur forest disease virus, JEV—Japanese encephalitic virus, WNW—West Nile virus, YFV—Yellow fever virus, DENV-1—dengue virus serotypes 1, DENV-2—dengue virus serotypes 2, DENV-3—dengue virus serotypes 3, and DENV-4—dengue virus serotypes 4, ZIKV—Zika virus. (**D**) Ribbon representation of TBEV E protein 3D structure (PDB 1SVB) with specified region 247–254 aa (orange arrow) and the fusion loop (purple arrow). The molecular coordinates for the structural analysis were derived from the Protein Data Bank and rendered using UCSF Chimera molecular visualizer, version 1.15 [26]. (**E**) Alignment of region 240–256 aa of TBEV glycoprotein E and peptides recognized by mAb FVN-32 selected from phage libraries.

The specific domain recognized by mAb FVN-32 was defined using western blot analysis of various truncated analogs of glycoprotein E TBEV: rE containing 1–401 aa (domain I, domain II, and domain III), rED1+2 containing 1–301 aa (domains I and II), and rED3_301 containing 301–399 aa (domain III). It was shown that the mAb FVN-32 recognized truncated proteins including domains 1 and 2: rE and rED1+2 (Figure 2). Therefore, the epitope recognized by mAb FVN-32 was localized on the DI and/or D2 glycoprotein E of TBEV.

To confirm and clarify localization of the epitope bound by mAb FVN-32, truncated variants of the region I + II were produced in bacterial cells. *E. coli* cell lysates containing rED2ΔA (1–141 aa), rED2ΔB (aa 130–195), rED2ΔC (aa 189–260), and rED2ΔD (aa 231–260) were employed in western blot analysis with mAb FVN-32. The mAb revealed truncated proteins rED2ΔC and rED2ΔD and did not recognize other variants. Thus, it was determined that the neutralizing epitope was in the region aa 231–260 of TBEV glycoprotein E.

For the epitope mapping, the phage display method was applied using combinatorial peptide phage libraries PhD-12 and PhD-C7C (New England Biolabs, Ipswich, MA, USA). The PhD-12 phage library is a population of filamentous bacteriophages, each of which carries an insertion of a random peptide from 12 aa within the minor surface protein p3; phage library PhD-C7C contains a population of M13 phages, each carrying a random peptide of 7 aa flanked by cysteine residues, which leads to the formation of a CxxxxxxxC loop on the p3 protein surface. Before each round of biopanning, both libraries were independently depleted by incubation with non-specific mAb 3G11, having the same IgG1/kappa subclass as the mAb FVN-32, to eliminate phages binding to the constant domains of mouse antibodies.

The presence of an inserted peptide in the gene encoding the phage protein p3 was analyzed by PCR using primers pIII_93. The DNA fragments encoding the selected peptides were sequenced; the deduced amino acid sequences of the peptide were compared with the amino acid sequence of the C-terminal fragment of domain II of TBEV glycoprotein. As a result, four different peptides with clear similarities to the sequence of the expected C-terminal fragment of domain II (aa 231–260) were selected from the library PhD-12 (Figure 2). Two different peptides were selected from the PhD-C7C library by binding them to the FVN-32 antibody. All of the selected peptides were mapped to the C-terminal fragment sequence of domain II, at aa 247 to 254 (Figure 2). This region is located between a short I-strand (aa 241–247) and short J-strand (aa 250–256) and formed a small lateral loop in DII of glycoprotein E. Three-dimensional modeling indicates that the identified target site for mAb FVN32 does not contact with the fusion loop (Figure 2).

## 4. Discussion

In this study, a new high-affinity mouse mAb FVN-32 against glycoprotein E of TBEV was selected and examined. It was shown that mAb FVN-32 had weak protective efficacy when administered one day post-exposure after challenge with of TBEV, strain Sofjin. The mean survival time of TBEV-infected animals from the test groups was similar to or better than that of each group of controls, despite the dose of the mAb. These results suggested that mAb FVN-32 did not enhance the infection in animals. Although this FVN-32 antibody has a high affinity constant, comparable to the *K_d_* of the previously described high-potency antibody FVN-145 [20], it has shown weak protective activity.

We defined that mAb FVN-32 recognized glycoprotein E domain DII and the neutralizing epitope was located in a fragment between aa 247 and 254. This region is highly conserved among TBEV-like orthoflaviviruses; corresponding sequences of other flaviviruses, especially mosquito-borne orthoflavivirus, slightly differ (Figure 2). In the tertiary structure of glycoprotein E, this small loop is located near the fusion loop, a site in the DII domain, which is highly conserved among different flaviviruses and plays a key role in virus penetration into the host cells. It has been previously shown that flavivirus-neutralizing antibodies with ADE were fusion-loop-specific or were targeted against various epitopes on DII [27,28,29,30,31,32,33].

The efficiency of antibody-mediated neutralization of flaviviruses depends on the affinity of the antibody and the availability of the epitope recognized by the antibody. In turn, the availability of the recognized epitope mainly depends on the structural heterogeneity of the molecules within the virion. This explains why the most effective virus-neutralizing antibodies are often direct to highly accessible epitopes, whereas weakly neutralizing antibodies tend to bind cryptic epitopes, which become available only during the infection of host cells. Among flaviviruses, accessible surface epitopes exhibit the highest degree of variability, which is why the most powerful neutralizing mAbs are often able to bind only a certain virus. Meanwhile, antibodies that promote ADE tend to be target cross-reactive epitopes [34,35].

We assume that a probable mechanism of virus neutralization by mAb FVN-32 interferes with conformational changes in the viral envelope, preventing the insertion of the fusion loop into the endosome membrane during viral infection of the cell as a result of binding of the mAb FVN-32 to its epitope. A neutralization mechanism similar to that of FVN-32 was observed for Fab 19/1786 [28]. It is known that antibodies blocking the release of the fusion loop are able to neutralize the virus on the one hand, and cause ADE on the other. However, mAb FVN-32, unlike anti-fusion loop antibodies, does not increase TBEV infection in model animals. Taking into consideration that anti-DII antibodies are mainly induced in vaccinated people [10], weakly neutralizing antibodies such as mAb FVN-32, which do not elicit ADE, ensure the success of vaccination against TBEV.

## 5. Conclusions

In this study, a new mouse mAb FVN-32 against glycoprotein E of TBEV was selected and characterized. In the animal model, mAb FVN-32 showed weak but clear protective efficacy (lower than 38%), being administered one day after mice infection with TBEV. Notably, the mean survival time in the antibody-treated groups of mice did not decrease compared to the mean survival times in the control group of untreated mice and the isotypic control group. This indicates that mAb FVN-32 does not enhance infection caused by TBEV in the used animal model. The epitope, recognized by mAb FVN-32, was localized in DII of the TBEV glycoprotein E using a panel of recombinant truncated fragments of the protein. The target site was mapped in a region between 247 and 254 aa on DII domain, which is conserved among orthoflaviviruses from the TBEV-group. This region forms a small lateral loop in DII of glycoprotein E and does not contact with the fusion loop. When mAb FVN-32 binds this lateral loop, this probably leads to changes in conformation of glycoprotein E that prevent exposure of the fusion loop. As a result, the attachment of TBEV to host cells decreases and this explains the protective efficacy of mAb FVN-32.

## Figures and Tables

**Table 1:** Primers used for the development of recombinant variants of DI and DII of TBEV glycoprotein E.

Truncated Protein E	Primer Name	Primer Sequences	Amino Acids
rED2ΔA	rED2ΔA_U	5′-CCGTTGGATCCATGGCCTCACGGTGCACACATCTGG-3′	1–141
	rED2ΔA_L	5′-GGCTTGAATTCCCGACTGTGTACACAATTTTGTTAGCGTCATACA-3′	
rED2ΔB	rED2ΔB_U	5′-CCGTTGGATCCGTGTATGACGCTAACAAAATTGTGTACACAGTC-3′	130–195
	rED2ΔB_L	5′-GGCTTGAATTCCCCTGAGCAAGGTCAACACCGCTGGC-3′	
rED2ΔC	rED2ΔC_U	5′-CCGTTGGATCCAGCGGTGTTGACCTTGCTCAGACC-3′	189–237
	rED2ΔC_L	5′-GGCTTGAATTCTTCCGCGTTGTTCCAATTCTGTGC-3′	
rED2ΔD	rED2ΔD_U	5′-CCGTTGGATCCGCACAGAATTGGAACAACGCGGAAC-3′	231–260
	rED2ΔD_L	5′-GGCTTGAATTCCCAGTCTGGTCTCCAAGGTTGTACACGTCC-3′	

## Data Availability

Not applicable.
